# Children’s failure to control variables may reflect adaptive decision-making

**DOI:** 10.3758/s13423-022-02120-1

**Published:** 2022-07-13

**Authors:** Neil R. Bramley, Angela Jones, Todd M. Gureckis, Azzurra Ruggeri

**Affiliations:** 1 Department of Psychology, University of Edinburgh, Edinburgh, Scotland; 2 Max Planck Institute for Human Development, Lentzeallee 94, Berlin, Germany; 3 School of Education, Technical University of Munich, Lentzeallee 94, Berlin, Germany; 4 Department of Psychology, New York University, New York, NY USA

**Keywords:** Causal sparsity, Causal learning, Interventions, Scientific reasoning, CVS

## Abstract

Changing one variable at a time while controlling others is a key aspect of scientific experimentation and a central component of STEM curricula. However, children reportedly struggle to learn and implement this strategy. Why do children’s intuitions about how best to intervene on a causal system conflict with scientific practices? Mathematical analyses have shown that controlling variables is *not always* the most efficient learning strategy, and that its effectiveness depends on the “causal sparsity” of the problem, i.e., how many variables are likely to impact the outcome. We tested the degree to which 7- to 13-year-old children (*n* = 104) adapt their learning strategies based on expectations about causal sparsity. We report new evidence demonstrating that some previous work may have undersold children’s causal learning skills: Children can perform and interpret controlled experiments, are sensitive to causal sparsity, and use this information to tailor their testing strategies, demonstrating adaptive decision-making.

**Supplementary Information:**

The online version contains supplementary material available at 10.3758/s13423-022-02120-1.

## Introduction

Imagine you are gifted some seeds for the very first time in your life: a little tomato plant! You want it to thrive, so you need to figure out what makes and keeps it healthy. How much sun, water, and fertilizer does it need? This kind of task requires performing a series of unconfounded experiments to isolate and control how the different variables under consideration (e.g., sun, water, and fertilizer) impact the system (e.g., the health of the plant). For example, one might keep the amount of sun and water constant, modify the amount of fertilizer, and see what happens. This approach—testing one variable at a time while holding all other variables constant—is often referred to as the *Control of Variables Strategy* (CVS: Kuhn & Brannock, 1977; Klahr, Zimmerman, & Jirout, 2011; Chen & Klahr, 1999). Mastering CVS is a crucial component of STEM curricula, featuring as one of the assessment criteria in national standards for science education (e.g., see National Academy of Sciences 2013 p.52). Indeed, STEM students are explicitly taught to make causal inferences using CVS (Kuhn & Brannock 1977; Klahr et al. 2011; Chen & Klahr 1999). However, previous work has suggested that children tend to manipulate multiple variables simultaneously when presented with problems like the one above, producing ostensibly confounded evidence (Wilkening & Huber 2004). Indeed, the education literature has generally taken a negative view of children’s spontaneous active learning abilities on the basis of experimental results, suggesting children struggle to acquire CVS without explicit instruction and extensive practice (reviewed in Zimmerman 2007; Schwichow, Croker, Zimmerman, Höffler, & Härtig, 2016), and only start to be able to transfer CVS training to new scenarios from around age 10 (Chen & Klahr 1999; Schauble 1996; Wilkening & Huber 2004; Kuhn 2007; Kuhn, Garcia-Mila, Zohar, Andersen, White, Klahr, & Carver, 1995; Klahr, Fay, & Dunbar, 1993; Klahr et al. 2011; Zimmerman 2007).

One interpretation of this is that children develop the cognitive competencies required for understanding and implementing appropriate active learning strategies only late in development (cf. Piaget 1977). On this view, children’s tendency to test multiple variables simultaneously reflects a general immaturity and lack of rigor in their scientific thinking. In this sense, it is considered part of the educators’ role to instill such scientific rigor in them by training them to implement CVS strategies.

However, we think there are nowadays good reasons to be skeptical of this account. The alternative interpretation that we explore here is that children’s observed failure in CVS tasks might stem from their bringing in different *assumptions* than those intended to be conveyed by cover stories like the one above, leading them to apply a different, yet ecologically effective, default strategy for active learning. As such, children’s failure in CVS tasks may depend on the fact that the task-relevant properties of the causal system were not conveyed with sufficient clarity for children to understand that a different strategy should be implemented, other than their default.

### When is CVS a poor strategy?

Several factors can (and should!) impact causal learning strategies, such as the functional form of the causes under investigation, their relationship, and whether the causal learning system examined is deterministic or stochastic (see Jones, Schulz, Meder & Ruggeri, 2018; Spiker & Cantor 1979; McCormack, Bramley, Frosch, Patrick, & Lagnado, 2016; Bonawitz, Denison, Gopnik, & Griffiths, Bonawitz et al. 2014; Horn, Ruggeri, & Pachur, 2016). Causal sparsity refers to the expected number of causally relevant variables in a system relative to the total number of variables. Mathematical analysis shows that expectations about causal sparsity mediate the effectiveness of different causal learning strategies, such that CVS is only sometimes the most effective approach (Coenen, Ruggeri, Bramley & Gureckis, 2019). In particular, as causal sparsity increases—that is, as the proportion of candidate causes expected to affect a given outcome decreases—manipulating a greater proportion of the variables at once can become dramatically more efficient than manipulating one variable at a time. For example, if we were engaged in finding a cure for a novel plant disease, it would be reasonable to expect that most things we might try will be ineffective. In this case, it is better to try several substances at a time until we observe an effect. In general, the most informative tests are those whose answers are expected to best narrow the learner’s hypothesis space. A particular manifestation of this is a “split-half” strategy (Nelson, Divjak, Gudmundsdottir, Martignon, & Meder, Nelson et al. 2014), which amounts to testing (as close as possible to) half the remaining causal variables with each intervention. This is optimal when there is known to be only one cause impacting the system, and all candidate causes are equally likely (Coenen et al. 2019).^1^

### Evidence for early competence in spontaneous active learning

Our initial skepticism about educational psychology’s negative perspective on children’s active learning ability stems from the growing number of studies demonstrating ways in which toddlers’ and preschoolers’ active causal learning skills are already quite sophisticated (Gopnik, Sobel, Schulz & Glymour 2001; Ruggeri, Sim, & Xu, 2017; Ruggeri, Swaboda, Sim, & Gopnik, 2019; Adams, Kachergis, Gunzelmann, Howes, Tenbrink & Davelaar, 2017; Lucas, Bridgers, Griffiths, & Gopnik, 2014; Cook, Goodman & Schulz, 2011; Kushnir & Gopnik, 2005; Schulz, Gopnik, & Glymour, 2007; McCormack et al. 2016). Toddlers and preschoolers have been shown to spontaneously make informative interventions to disambiguate the causal structure of a system, both in experimental settings and during spontaneous play Kushnir & Gopnik (2005); Cook et al. (2011); Sim & Xu (2017); Schulz & Bonawitz (2007), and the efficiency of these interventions has been shown to increase with age McCormack et al. (2016). Already by age 6, children demonstrate some ability to *identify* and *plan* controlled tests (Sodian, Zaitchik, Carey, 1991; Osterhaus, Koerber, & Sodian, 2015), and even preschoolers can be trained to use CVS as a domain-general strategy if given regular feedback and guidance (van der Graaf, Segers, & Verhoeven, 2015). More recent work shows that even 3- and 4-year-olds rely on a variety of exploratory strategies depending on the statistical structure of a task, *selecting* the more efficient strategy from among a set of options Ruggeri et al. (2019).

How can children be robust and effective causal learners but also fail dramatically at implementing CVS in scenarios where adult scientists deem it to be the appropriate testing strategy? On the one hand, this is in line with previous work showing it is hard to robustly change children’s information search strategies through instruction (e.g., question-asking strategies; see Courage 1989; Denney, Denney & Ziobrowski, 1973; Ruggeri, Walker, Lombrozo, & Gopnik, 2021).

However, the *spontaneous* adaptiveness of children’s active learning strategies has seldom been directly investigated outside of question-asking tasks. In causal learning, younger learners (4-year-olds) seem to be more flexible than older learners (6-year-olds; Gopnik & Bonawitz 2015) and even adults in correctly drawing inferences about unusual causal relationships from observation (Lucas et al. 2014). Moreover, preschoolers’ causal learning is already consistent with Bayesian principles at age 4 (Sobel, Tenenbaum, & Gopnik, 2004; Bonawitz et al. 2014). Together, these findings suggest that primary school children, just like adults, may be sensitive to context and able to adapt their learning strategies to the causal sparsity of a presented system.

## Experiment

In this paper, we seek to reconcile conflicting findings from the cognitive developmental and educational literature to explore whether children’s apparent failure to implement CVS may be due to their default assumptions about the tasks they are presented with. In particular, we focus on children’s sensitivity and ability to tailor their causal learning strategies to the *causal sparsity* of the system they are investigating. To test this hypothesis directly, we opted to depart from the implicit complexity of naturalistic cover stories and focus on a mathematically clean, assumption-transparent setting. We presented 7- to 13-year-old children with an unfamiliar ‘box-of-switches’ and asked them to determine how it worked. This age range was motivated by prior research suggesting a strong developmental shift in children’s information search strategies between the ages of 7 and 13 Ruggeri & Katsikopoulos (2013); Ruggeri & Feufel (2015); Ruggeri & Lombrozo (2015); Mosher & Hornsby (1966). Additionally, piloting suggested that children younger than 7 failed to understand the instructions and affordances of the switch-box task. We manipulated children’s expectations about the causal sparsity of the system and measured if this changed how they approached the problem, with a particular attention to the spontaneous use of CVS.

### Methods

#### Participants

Participants were 53 7- to 9-year-olds ($M = 8.19$ years, SD = 0.59, 24 female) and 51 10- to 13-year-olds ($M = 11.17$ years, SD = 1.28, 16 female) recruited and tested in museums in [blind for review]. The sample size was chosen based on a simulation-based power analysis. This was based on a conservative estimate of the effects found in previous work with Fisher’s exact test—i.e., a difference of 40% between participants’ strategic approach to the different causal sparsity conditions, compared to the 66% difference found in a related study with adults (Coenen et al. 2019)—and indicated a sample of about 50 participants per age group (*N* = 25 per condition) to achieve 80% power with $\alpha$ = .05. All participants were [blind for review] or fluent in [blind for review]. IRB approval was granted and informed consent was obtained from parents prior to children’s participation.

#### Design and materials

**Fig. 1:**
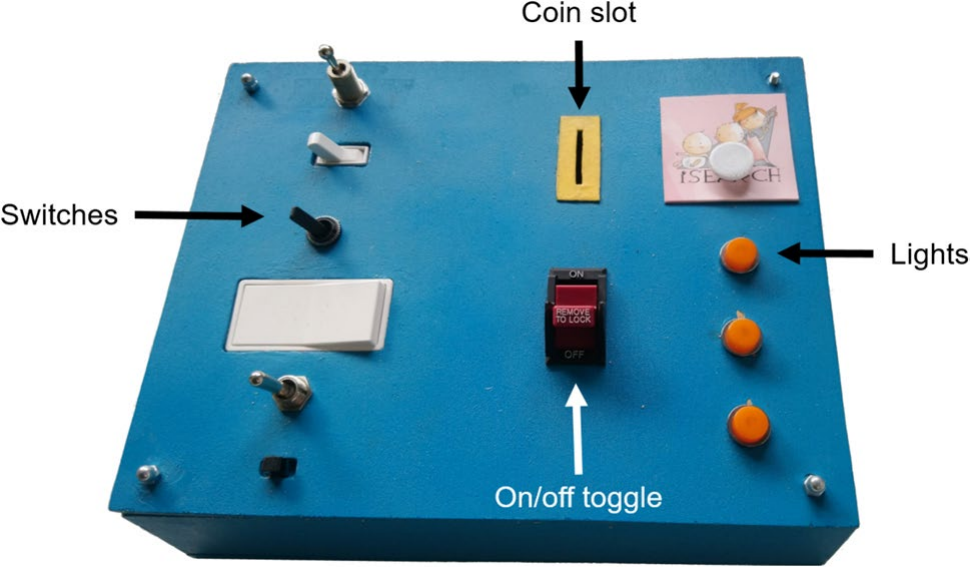
Annotated photograph of switch box used for the study

Participants were presented with a wooden box measuring approximately $35\times 25\times 10$cm. The top of the box featured six different switches on the left side (corresponding to the six putative causes), three lights (outcome), a red activation toggle and a slot to insert coin tokens (Fig. 1). We limited the number of switches to six as the number of variables to be considered in a causal learning task is known to impact children’s ability to use CVS successfully (Wilkening & Huber 2004). We wanted children to be able to complete the task without assistance, and we wanted to minimize the impact of working memory on task performance.

The box was initially inactive, and while it remained inactive the lights would never turn on irrespective of how the switches were set. It could be activated by putting a coin in the coin slot and then pressing activation toggle whereupon the lights would turn on if at least one working switch was in the on position. The box contained a raspberry Pi microcomputer (Richardson, & Wallace, 2012) that determined the outcomes and recorded children’s actions during the study. Participants were randomly assigned to two conditions: s*parse* and *dense*. In the sparse condition, children were told that only one of the switches worked. In the dense condition, they were told that only one of the switches was broken—that is, all the switches could turn on the lights, except for one. A single working switch in its “on” position was enough to make the lights come on when the activation toggle was pressed. In both conditions, children’s task was to find the one working or broken switch. Which switch was working or broken was randomly determined for each child. Participants therefore set the switches in different positions, then paid a coin to turn on the activation toggle and see whether the lights would turn on.

#### Relation to traditional CVS tasks

Like most traditional CVS tasks, our switch box task requires participants to determine which variables affect the outcome. This involves generating a set of hypotheses to consider, then intervening to test these hypotheses, observing the outcome, and coming up with a new intervention to distinguish between the remaining possibilities. As with traditional CVS tasks, there is the danger of causal overshadowing leading to confounded interventional evidence (Waldmann 2001). In our case, if a learner turns on multiple switches and sees the outcome occur, this does not tell them whether one or multiple of these switches was causally responsible. However, our task also differs from classic CVS tasks in several respects. First, it includes a sparsity manipulation, which has never been explicitly varied in classic CVS paradigms. Second, our variables of interest and outcome are binary, while CVS tasks typically include continuous variables and outcomes or variables which can take a continua of states (e.g., ramp length or texture as variables, and distance traveled by a ball as the outcome; Siler, Klahr, Magaro, Willows, & Mowery, 2010). However, these continuous variables are introduced qualitatively such that the size and stability of each effect is left unspecified. This is why any choices that change any more than one input value relative to any earlier test are considered to be confounded. Our binary disjunctive setting means that a CVS strategy manifests a little differently than in these classic tasks. Controlling variables is achieved by leaving them turned off in a test, rather than by leaving them in whatever position they were in in an earlier trial, however the deeper principle is identical.

Crucially, CVS *is not* the most effective way to solve our task in the Sparse condition. However, it is the only effective method of doing so in the dense condition, and it still is a valid approach in the Sparse condition, just sub-optimally effective. Our goal in this paper is thus to examine children’s active causal learning performance and use the results to reassess the question of what children’s previously documented difficulties with CVS tells us about their active learning abilities, default assumptions about the task characteristics, and strategy flexibility.

#### Procedure

Children were first familiarized with the box and its components. The experimenter explained the binary (left = off, right = on) nature of the switches and the difference between broken and working switches. Children were then instructed that they had to identify the working switch (in the Sparse condition) or the broken switch (in the dense condition). In both conditions, before starting the task participants were led by the experimenter through two familiarization trials to practice the procedure and experience both outcomes. All the switches were initially set to their “on” position. The experimenter pointed this out, then activated the box using the main activation toggle, causing the lights to turn on. Next, the experimenter set all the switches to their “off” position, one by one, and again activated the box using the main activation toggle, this time demonstrating that the lights did not turn on.

At this point, control was handed to the child and they were asked to identify the target switch. Children could then test any combination of on/off switches and see if the lights turned on as a result. All switches were set to the “off” position again by the experimenter before the beginning of each new trial and the child could then turn on any combination they liked before activating the machine again. To promote efficient search, participants were given six tokens at the beginning of the experiment, and had to pay one token using the slot provided (see Fig. 1) every time they wanted to test a new switch combination. Participants could therefore perform up to six tests, but could stop at any time before then if they felt they had found the target switch. At that point, they were then asked to indicate which switch they thought was broken/working. The experimenter tested this by turning that switch on and activating the box so they could observe the outcome. If the child’s selection was correct, the lights would come on in the Sparse condition or not come on in the dense condition, the experiment ended and they could keep their remaining tokens (each worth 0.50€). If not, they were given the option to perform more tests and guess again, or guess again right away, until they found the correct switch. The maximum reward was thus 2.50€, achievable if they were lucky enough to reach the solution after a single test trial. By following the ideal “split-half” strategy it was possible to achieve $\approx 1.90$€ on average in the Sparse condition, while in the dense condition, the only effective strategy was to test one switch at a time, with an expected return of 1.25€. If they used up all their tokens, or got the answer wrong, children received a sticker as a compensation reward.

Task instructions, analysis code and data are available on the Open Science Framework.

### Results

#### Analysis of the first intervention

The number of switches tested in the very first intervention is crucially indicative of the way children approach the task in the different conditions. The number of children who tested one or multiple switches in each condition is shown in Table 1. We used Bayesian logistic regression to evaluate whether age group or condition influenced the tendency to test one versus multiple switches in the first intervention while also allowing as to assess support for the nulls if required (See S1. for detailed parameter settings and sensitivity analyses). Testing multiple switches was more common in older children (51 vs. 28%) and in the sparse condition (50 vs. 29%). Both age group ($\mathrm {odds~ratio~[OR]} = 2.24$, $95\%~\text {credible~interval}~[95\%\text {CI}] = [1.05, 4.9]$, Probability of Direction [PD] = 98.09%, $\text {Bayes~factor~[BF]}=3.44$) and condition ($OR = 0.47, 95\%CI = [0.22, 1.01]$, PD = 97.25%, $BF=2.34$) appeared to affect the proportion children testing a single switch in the first trial, but the data did not suggest that age group and condition interact ($OR = 0.97, 95\%CI = [0.3, 3.18]$, $PD = 52.30\%, BF=0.57$), with the BF<1 suggesting anecdotal evidence against its existence (Jeffreys 1961). This suggests age and condition contributed independently to children’s propensity to test one switch with their first intervention such that children in both age groups were similarly sensitive to causal sparsity, while their default approach seemed to shift with age from testing one to testing multiple switches at a time. However, the size of the effects looking only at the first test were relatively modest. Indeed, 3.3% of the posterior for age group and 5.3% of the posterior for condition falls within the region of practical equivalence with the nulls of no effect (ROPE, Kruschke et al. 2018).^2^ We then analyzed children’s sequences of interventions in more detail.

**Table 1:** Counts and percentage of children testing one or multiple switches on first intervention

Age group	Condition	Test one (first trial)	Test multiple (first trial)
Younger	Sparse	15 (62.5%)	9 (37.5%)
	Dense	23 (79.3%)	6 (20.7%)
Older	Sparse	11 (39.3%)	17 (60.7%)
	Dense	14 (60.9%)	9 (39.1%)

#### Strategy use

Twelve children were excluded from subsequent analyses because their intervention data was incomplete due to technical difficulties, leaving 92 participants for whom we have a complete record. In total, 46 7- to 9-year-olds ($M = 8.21$ years, $SD = 0.55$, 20 female) and 46 10- to 13-year-olds ($M = 11.18$ years, SD = 1.34, 12 female) were included in the following analyses.

We classified children’s strategies into three types based on how many switches they turned on in each trial. For this, we focused on the trials in which there were at least four switches still in contention (216/283 trials). These were the trials in which testing multiple variables simultaneously was more effective than testing any one variable in the sparse condition:^3^

**Fig. 2:**
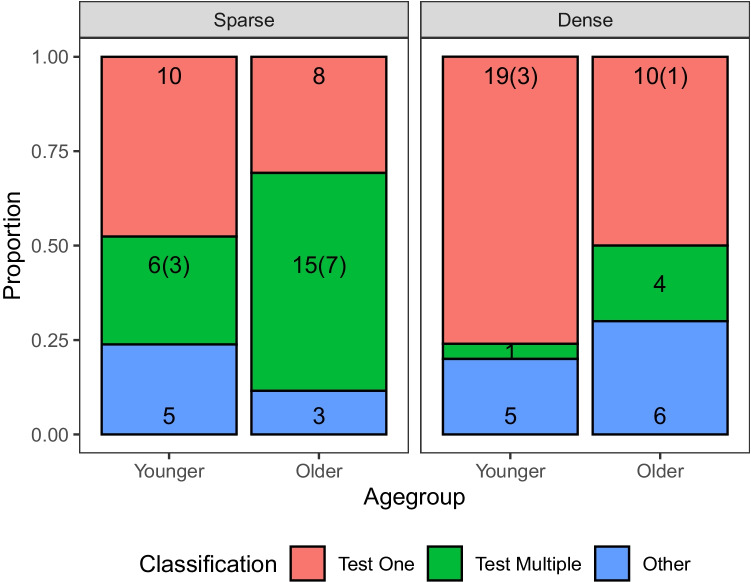
*Bars* show proportion of children in each age group classified as using each strategy in each condition. *Numbers* show the number of children in each bar and *bracketed numbers* show subset whose choices were additionally information-optimal across all the trials used in the strategy classification

*Test one* denoted strategies in which exactly one switch was flipped on in each test. *Test multiple* denoted strategies in which more than one switch was flipped on in each test. Strategies which did not fit either of these criteria were classified as *other*. The Other classification included children who switched back and forth between a test one and a Test multiple strategy, but also children who started with a test multiple strategy before switching to Test one, or vice-versa. The proportion of children who used each strategy is shown in Fig. 2.

An ideal information-gain-maximizing learner would follow a *Test multiple* strategy in the sparse condition, specifically testing 2–4 switches with their first switches and exactly half of those still in contention with their second test (rounding up or down if this is an odd number). Meanwhile, an ideal participant in the dense condition would follow a *test one* strategy and furthermore choose a new switch to test with each test.

As with the analysis of the initial intervention, we used Bayesian logistic regression to model whether age group or condition impacted on strategy classification. Bolstering our analyses of the first intervention, we found that older children were less likely to employ a test one strategy ($OR = 0.45, 95\%CI = [0.2, 0.99], PD = 97.7\%, BF = 2.95$), and that test one was significantly more common in the dense condition ($OR = 2.41, 95\%CI = [1.1, 5.35], PD = 98.7\%BF = 4.33$). The data did not suggest that age group and condition interacted $OR = 0.83, 95\%CI = [0.25, 2.75]$, $PD = 62.0\%$, $BF = 0.66$. Older children were also more likely to employ a consistent Test multiple strategy ($OR = 2.93, 95\%CI = [1.21, 7.38]$, $PD = 99.2\%, BF = 7.2$), and this strategy was substantially less common in the dense condition ($OR = 0.23, 95\%CI = [0.09, 0.58]$, $PD = 99.9\%, BF = 55.8$). Again, the data did not suggest an interaction between age group and condition ($OR = 0.88, 95\%CI = [0.23, 3.3], PD = 57.5\%, BF = 0.70$).

Strikingly, 17/19 (89%) of the older children who classified as test multiple guessed the correct switch, while only 3/7 (43%) of younger participants classified as test multiple did so (Fisher’s exact test, $p=.003$; Bayesian contingency analysis, BF $= 7.7$). In the sparse condition, these proportions were 15/15 (100%) and 2/6 (33.3%), respectively (Fisher’s exact test, $p = .002$, BF = 95). Thus, together with our analysis of children’s first intervention, these results suggest that all children were sensitive to causal sparsity, although only older children were able to learn effectively from the tests performed. This is consistent with several recent studies that find the ability to make reliable causal inferences develops separately, and indeed lags behind, the ability to perform appropriate interventions (Nussenbaum, Cohen, Davis, Halpern, Gureckis, & Hartley, 2020; Bramley & Ruggeri, 2022; Meng, Bramley, & Xu, 2018).

#### Performance

In the sparse condition, 62% of younger participants (13/21) and 88% of older participants (23/26) identified the correct switch, having made a respective $M = 3.0$ ($SD = 1.5$) and $M = 2.8$ ($SD = 1.3$) interventions. In the dense condition, 64% of younger participants (16/25) and 50% of older participants (10/20) identified the correct switch, having made a respective $M = 3.5$ ($SD = 1.3$) and $M = 3.0$ ($SD = 1.5$) average interventions. Bayesian proportion analysis tests (Morey, & Rouder, 2011) comparing against an “eyes closed” chance accuracy level of 1/6, yielded Bayes factors >30 in all conditions.

Bayesian logistic regressions predicting performance were practically indeterminate, with marginal anecdotal support for the null of no age group effect $OR = 1.32, 95\%CI = [0.59, 3.01], PD = 74.6\%, BF = .50$, slight support for an effect of condition $OR = 0.49, 95\%CI = [0.21, 1.11], PD = 95.7\%, BF = 1.67$ and some for an interaction $OR = 0.37, 95\%CI = [0.11, 1.23], PD = 94.8\%, BF = 2.33$. In no case did the credible interval of odds ratios exclude 1. Bayesian Poisson regressions of the number of trials and the number of guesses children made with age group and condition as predictors also showed no meaningful effects (see Supplementary Materials S2).

#### Expected information gain of children’s selections

The effectiveness of children’s interventions can also be explored using expected information gain (EIG). EIG is a common measure for how valuable information-seeking actions are to a learner, given their current state of uncertainty and learning goals (Nelson 2005). A detailed explanation of how EIG is calculated here can be found in Supplementary Materials S3. Here, the relative values of the available interventions are partly a function of learning condition. The sparse condition has a wider range of actions that are potentially informative: Any combination of between 1 and 5 switches is informative on the first test and many continue to be informative as the space of possibilities is narrowed, but within these options, choices that more evenly divide the remaining options are more informative than those that do so unevenly. In contrast, in the dense condition only a smaller range of interventions is informative—only those that turn on a single switch and have not already been performed.

**Fig. 3:**
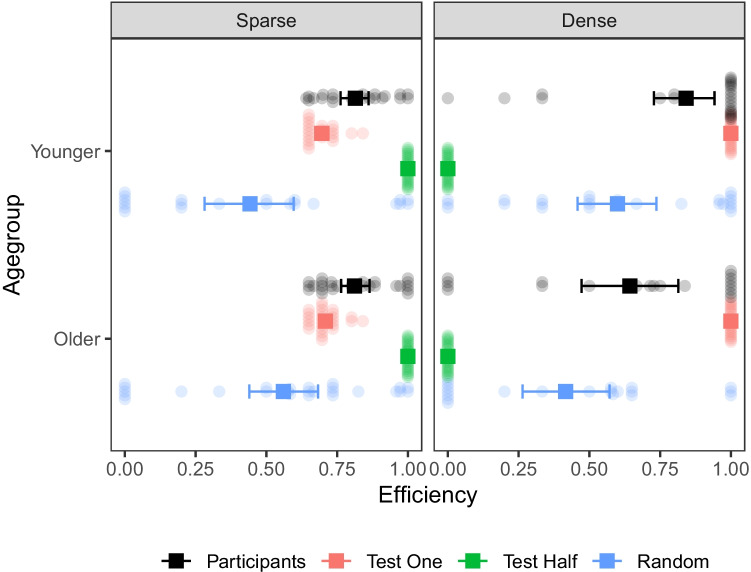
Efficiency of interventions relative to optimal choice. *Black* = participants, *red* = Simulated pure test one learners, *green* = Simulated pure Test half learners,* blue* = Simulated random interveners. *Squares* and *error bars* show group means± bootstrapped confidence intervals, and translucent points show individual participant averages

To account for these differences, we computed the efficiency of each participant’s interventions as a proportion of the most informative intervention available at that point from the perspective of an optimal learner that maximizes EIG at each step of the search process, accurately integrating the evidence from all the previous interventions. As baselines for comparison, we also simulated a set of learners that chose each intervention at *random*, flipping switches on with $p=.5$ but performing an equivalent total number of interventions as the participants. We also simulated pure *test one* learners, that always turned on one of the remaining untested switches with each new intervention and pure *Split-half* learners who always turn on half the remaining possibly-working switches. Figure 3 shows the efficiency of participants’ interventions compared to those of the simulations.

We used Bayesian beta regression to assess whether efficiency differed between age groups and conditions. Efficiency did not appear to depend on age group $OR = 0.86, 95\%CI = [0.65, 1.16], PD = 83.75\%, BF = 0.24$ or condition $OR = 1.03, 95\%CI = [0.77, 1.38], PD = 57.7\%, BF = 0.15$, but the data was consistent with an interaction such that older children performed worse in the dense condition $OR = 0.68, 95\%CI = [0.46, 0.99], PD = 97.8\%, BF = 1.45$. We then asked whether participants’ interventions were more efficient than those of simulated random interveners, including age group condition and their interaction as covariates.^4^ This reveals that participants’ interventions were more efficient than random choices $OR = 0.52, 95\%CI = [0.42, 0.64]$, $PD = 100\%, BF>1000$. Focusing on the sparse condition, we can ask if participants were additionally more efficient than simulated test one learners; 62% of participants were more efficient than Test one and a further 26% were equally efficient, while only 13% were less efficient. A Bayesian beta regression, including a *participants vs. test one’* factor shows a clear advantage for participants over simulated test one learners $OR = 0.77, 95\%CI = [0.7, 0.85]$, $PD = 100\%, BF>1000$ as well as support for the nulls with respect to there being any main effect of age group $OR = 1.04, 95\%CI = [0.84, 1.28]$, $PD = 63.4\%, BF = 0.12$ or interaction $OR = 0.96, 95\%CI = [0.79, 1.17]$, $PD = 65.4\%, BF = 0.11$. A more detailed analysis of children’s strategy efficiency, which takes into account early stopping and unnecessary tests, is presented in S4.

## Discussion

We investigated to what extent 7- to 13-year-olds can perform efficient causal interventions and learn from them without guidance. In particular, we examined whether and how children adapted their learning strategies to contextual knowledge about the causal sparsity of the system under investigation. We found that children did indeed intervene differently depending on the context they were presented with, being more likely to test multiple switches when they expected one switch to work, and test one switch at a time when they expected many to work. Thus, we show that in a setting with clear instructions, and consequently transparent background assumptions, children can implement a test one approach when it makes sense to do so. Our findings additionally suggest that children’s default active causal learning strategy may actually shift with age *from* testing causal relationships one at a time, *towards* testing multiple causal relationships simultaneously, with a greater proportion of younger children testing one switch at a time than older children in both conditions. Both these findings challenge the educational literature on CVS (Kuhn et al. 1995; Klahr et al. 2011), which has argued that children tend to manipulate multiple variables at once even when they should not Wilkening & Huber (2004), and require extensive training and instructions to eventually override this tendency. We think these results line up better with the idea that children are effective active learners from an early age (cf. Gopnik et al. 2001; Ruggeri et al. 2017, 2019; Lucas et al. 2014; McCormack et al. 2016).

In one condition of our task, testing multiple causal relationships simultaneously resulted in completely confounded evidence, while in the other, testing one at a time resulted in less evidence per test, and lower rewards (recalling children made 0.50€per remaining token, if correct). Both younger and older children showed similar amounts adaptivity, being more likely to test one switch at a time when they had reason to believe the system in question was dense enough to necessitate this and more likely to test multiple switches at a time when it was advantageous. However, both age groups also exhibited some resistance, with some children still testing multiple variables at a time the dense condition and some still testing one at a time in the sparse condition. Our suggestion is that children’s tendency to test multiple variables should not be taken as evidence that they are poor active learners, but rather indicate that they have learned a useful default strategy that is not CVS. Indeed, the value of testing multiple is critical in domains like question asking, and becomes increasingly powerful as children become more able to reason about larger numbers of hypotheses. We thus suggest that the educational psychology community should think more carefully about why children struggle to perform unconfounded experiments in CVS paradigms while succeeding performing appropriate interventions in other tasks and domains.

One explanation for the incongruity that we find persuasive is that the ecological assumptions built into our task are a closer reflection of the generic active causal learning contexts that children face, compared to those in CVS tasks. The idea that the world is causally sparse has been floated a number of times in the cognitive science literature (Oaksford, & Chater, 1994; Lu, Yuille, Liljeholm, Cheng, & Holyoak, 2008). Favoring sparse solutions is also a standard regularization principle for causal inference in statistics (Glymour, Zhang, & Spirtes 2019). As such, a *ceteris paribus* assumption that any given action is unlikely to produce a desired effect might lead to the emergence of a default strategy to test enough potential causes so that the outcome of one’s test is maximally uncertain (lining up with “split-half” in our sparse condition). Of course, to succeed in the world, one must learn to apply strategies in a context-sensitive way. As one learns more about the world, one can use prior knowledge to select and test only variables that have a good chance of playing a role in the outcome considered, so changing the sparsity of the resulting active learning problem. Arguably, in the standard CVS setting, the variables involved fit this bill, having a relatively high probability of affecting the outcome under a mature understanding of the scenario, but it also seems plausible that this prior would be much stronger in adults, with at least a high-school level understanding of the biological and physical mechanisms used as scenarios. Our assumption-transparent box task minimizes the influence of such priors and so provides a more transparent window on children’s active learning.

Besides often only tacitly implying a causally dense environment, CVS tasks are also set up such that it does not matter what values the other variables are held at, as long as they remain fixed while the putative cause is manipulated. This implies that the variables are not interacting. We agree that, if this holds, it would be appropriate to follow CVS. However, we disagree this is common, even for dense environments. Returning to our initial example, as adults, we know that no amount of fertilizer will impact the health of a plant it if is not also given at least some water. This means that a pure CVS user, who manipulated fertilizer while fixing water to zero, would fail to discover its causal role. More generally, in situations where the possibility of interactions and arbitrary functional relationships is to be considered, a more complex, rich and exhaustive approach than CVS would be required to definitively identify the causal structure.

In the worst case, one would have to test all the level combinations of all variables to ensure one has not missed a causal influence that manifests only under particular settings of other variables. We mention this to highlight that there are other relevant tacit assumptions about the causal environment under consideration that determine whether and when CVS is sufficient as a causal discovery strategy.

The developmental trajectory we found here is extremely similar to that of children’s question-asking strategies. In 20-Questions paradigms, children under the age of 7 almost exclusively test one hypothesis at a time (e.g., “is it this one parrot?”). Between the ages of 7 and 10, children begin to ask questions that target several hypotheses at once (e.g., “is it a bird?”), until this becomes the default strategy in adulthood (Mosher & Hornsby 1966; Ruggeri & Feufel 2015; Herwig 1982). This parallelism suggests that children’s learning strategies may reflect their learning abilities, broadly progressing from an ability to consider and reason about only one hypothesis at a time to being able to consider a range of hypotheses and their relationship with the outcome. Indeed, children’s ability to update multiple entries in working memory improves with age (Pailian, Carey, Halberda, & Pepperberg, 2020). On this view, it is plausible that younger children in our study may have favored Test one in part because a Test multiple strategy was too resource-intensive rather than because it guards against confounded experiments (as it is motivated in the CVS literature).

An additional possible explanation for the developmental shift toward test multiple in children’s default strategies could be that older children brought stronger prior assumptions to the task—e.g., about how parallel and serial circuits might work—and that these conflicted with the disjunctive behavior of the switch box in the dense condition.

This might help explain the puzzling pattern that younger children performed slightly better than older children in the dense condition. That is, it could be that older children’s prior beliefs “drowned out” the context given by the instructions. Consistent with this interpretation, some older children persisted with a Test multiple strategy in this condition, even though it was ineffective.

To conclude, our results suggest that previous conclusions—that children have inherent difficulties and need extensive instruction to master the Control of Variables Strategy—may have undersold children’s causal reasoning and strategic abilities. Children, at least under the conditions investigated in this paper, demonstrate not only the ability to plan, perform and interpret controlled experiments without guidance, but also the flexibility and adaptiveness required to shift their reliance on different hypothesis-testing strategies depending on the causal sparsity of the system under investigation. Designing a good experiment requires an understanding of the structure of the problem one wants to learn about (cf. Crupi, Nelson, Meder, Cevolani & Tentori, 2018). In this sense, no learning strategy is *always* best—not even CVS, a fact that might come as a surprise even to professional scientists.

Our findings highlight the crucial importance of considering children’s sensitivity to context, and consequent appropriateness of different strategies when teaching STEM subjects and scientific thinking. Children’s ecologically reasonable prior beliefs may account for some resistance to applying a certain strategy to the problems they are presented with. This is especially true if care isn’t taken to get across the assumptions that warrant that specific strategy. It may be worthwhile to consider providing children with a toolbox of strategies and teaching them how and when to use each one, rather than focusing on training them to use and master one strategy in particular, which may fail them in many situations.

## Supplementary Information

Below is the link to the electronic supplementary material.

Supplementary file 1 (pdf 1668 KB)

## Footnotes

1 As well as being inefficient in sparse settings, CVS is also insufficient in settings in which causes interact. We focus on the former limitation here expand on the latter issue in the General discussion.

2 ROPE measures the proportion of the posterior density for an effect that falls within the 95% credible interval of what one would anticipate finding under the null of no effect.

3 With three potentially working switches left, testing one or two of these three is equally informative.

4 Since each simulation was paired to a participant in terms of the ground truth and number of tests performed, we also included a random effect for subject ID.
