# Supplementary material for: Children’s failure to control variables may reflect adaptive decision-making
Source: Psychon Bull Rev. 2022 Jul 13;29(6):2314–24. doi: 10.3758/s13423-022-02120-1 (PMC9722808; doi:10.3758/s13423-022-02120-1)
Supplement: Supplementary file 1 — (pdf 1668 KB) [file 13423_2022_2120_MOESM1_ESM.pdf]

Supplementary Materials

S1. Bayesian Regression Analyses

We used R's `brms` package for all Bayesian regression analyses (Bürkner, 2017). We chose a normal prior for all fixed effects and used default priors for intercept and interaction terms. For all analyses taking P(Correct) as dependent variable via logistic link function, we used Normal(0,1) prior for log odds ratio coefficients, such that under the prior, a 2.7 fold increase or decrease in the chance of answering correctly would equate to 1 Standard Deviation. We found default `brm` sampling settings resulted in unstable estimates in some cases, particularly for Bayes Factors. Therefore we doubled the number of MCMC chains from 4 to 8 and increased the length of the chains fivefold from 2,000 to 10,000 iterations, finding this greatly improved stability. We report estimates, 95% credible intervals following (Kruschke, 2013) and for comparison with traditional p values we include Probability of Direction (PD) statistics (Makowski, Ben-Shachar, Chen, & Lüdecke, 2019) and comment on Regions of Practical Equivalence (ROPE) with the nulls (Kruschke, 2018). We also performed a sensitivity analysis for key analyses varying the prior between a strong prior expectation for the null Normal(0, 0.2) and a diffuse Normal(0,5). The full analysis pipeline is included in the OSF Repository.

We also fit Bayesian regressions to predict intervention efficiency relative to optimally efficient choices. Since this constitutes proportion data, we model it using a Bayesian beta regression. However, this is complicated by the presence of proportions of exactly zero (5 of 92) and one (17 of 92). Beta regression requires data to be in $(0, 1)$ rather than $[0, 1]$. Thus, we followed Smithson and Verkuilen (2006) and used the correction $x' = \frac{x \times (N-1) + \frac{1}{2}}{N}$ as a principled re-scaling of the proportions before fitting the model. Conceptually this incorporates a prior of $\frac{1}{2}$ on each participant's proportion, and so accommodates that some proportions are based on more trials than other, with most zeros and ones occurring for participants who performed a small number of interventions in total. For example, if a participant performs only two interventions, but both are maximally efficient, their proportion is adjusted from 1 to $\frac{1 \times 1 + \frac{1}{2}}{2} = .75$, while

For Review Only

four maximally efficient interventions would lead to 0.875.

To measure whether accuracy was statistically above chance in each condition, we used `proportionBF` from R's `BayesFactor` library (Morey & Rouder, 2011). To test whether accuracy differed by age for participants characterised as Test Multiple, we used `contingencyTableBF` function from the same library. For this, we selected the 'jointMulti' sampling scheme under which total N is fixed, and observations are assigned to cells with fixed probability.

S2. Number of interventions and guesses by Age Group and Condition

Table S1 details Poisson regressions predicting number of interventionsa and guesses as a function of age group and condition.

Table S1

Poisson Regressions Predicting Number Interventions and Guesses by Age Group and Condition

	N Interventions			N Guesses		
	β	95% CI	Bayes Factor	β	95% CI	Bayes Factor
Intercept	2.99	2.33–3.78		1.29	0.89–1.82	
Age group (Younger)	0.93	0.67–1.3	0.18	0.83	0.51–1.36	0.25
Condition (Sparse)	1.17	0.85–1.6	0.25	1.11	0.7–1.76	0.32
Age group × Condition	0.91	0.58–1.44	0.25	1.39	0.73–2.64	0.52

Note: β coefficients and confidence intervals transformed to natural odds ratios. Reference groups for factors indicated in brackets

S3. Expected information gain calculation

In this task, the learner is confronted with a causal system with $N = 6$ binary independent variables, I, of which a subset of variables $C \subseteq I$ (i.e., individual switches) can affect the outcome when active (i.e., switched to the "on" position, and one binary outcome, o (i.e., the lights turning on). The probability of the outcome given a specific

For Review Only

setting of variables is

$$P(o = 1|C) = \begin{cases} 1, \text{if } \exists\ c \in C \wedge (c = 1), \\ 0, \text{otherwise.} \end{cases} \tag{1}$$

Simply put, the outcome occurs if, and only if, any of the variables in C are currently active. The learner must decide how to manipulate the variables to determine which are causally relevant. We assume that the learner's optimal strategy consists of choosing a switch setting, $s \in S$, which maximizes the *Expected Information Gain* (EIG) with respect to the system. EIG quantifies the expected reduction in uncertainty over the hypotheses H after having made an intervention on the system and observed an outcome. Here, the learner's hypotheses are possible sets of causally relevant variables, i.e., $H = \{C_1, ..., C_6\}$. Note that the contents of H differ between conditions because of the differences in sparsity. In the Sparse condition, each set (e.g., C_1) contains only one switch because only one switch can activate the lights, while in the Dense condition, each C contains a combination of 5 switches, as all but one switch can turn on the lights. We consider a simple case of binary outcomes ($o = 1$ or $o = 0$) with the likelihood of an outcome given by Equation 1. A learner's EIG is calculated as

$$\text{EIG}(s|H) = \text{SE}(H) - \sum_{j=0}^{1} P(o = j|s)\text{SE}(H|s, o = j), \tag{2}$$

where SE represents the Shannon entropy over a distribution of hypotheses (Shannon, 1951), which in this study are possible causes of the light turning on. The marginal likelihood of each outcome is then given by

$$P(o = j|s) = \sum_{i=1}^{6} P(o = j|C_i; s) \tag{3}$$

and the prior entropy (i.e., the uncertainty as to whether each candidate hypothesis is correct before a test) is

$$\text{SE(H)} = -\sum_{i=1}^{6} P(C_i) \log P(C_i). \tag{4}$$

After observing the outcome of a test, the learner's beliefs about each hypothesis are

For Review Only

updated following Bayes' rule,

$$P(C_i|o) = \frac{P(o|C_i)P(C_i)}{\sum_{j=1}^{6} P(o|C_j)P(C_j)}, \quad (5)$$

and the entropy over the updated set of hypotheses becomes

$$\text{SE}(H|s,o) = -\sum_{i=1}^{6} P(C_i|o)\log P(C_i|o). \quad (6)$$

S4. Early stopping and unnecessary tests

Stopping early and making unnecessary tests are two kinds of search errors that can provide additional insight into the quality of a learner's search. Stopping one's search before identifying the correct switch may occur if a participant searched inefficiently and runs low on tests and chooses to guess. However, guessing before it is advantageous to do so might indicate a misunderstanding of the task or application of an inappropriate strategy. Making "unnecessary tests" — that is, tests that occur after the target switch could have been identified and which therefore do not provide any additional information from a normative perspective — would suggest that children may find it difficult to keep track of the evidence gathered previously, or that they don't believe a trial fully rules out a switch setting.

The number and percentages of children stopping early and performing unnecessary tests is shown in Table S2. Performing unnecessary tests was rare, with only three children performing (either one or two) unnecessary tests. However, stopping early was common in both conditions for younger children and just in the Dense condition for older children.

Bayesian logistic regressions, predicting early stopping with Condition and Age Group, show that the odds of stopping early is higher in the Dense condition $OR = 2.69, 95\%CI = [1.22, 6.1], PD = 99.2\%, BF = 7.3$ but does not appear to differ by age group $OR = 0.67, 95\%CI = [0.3, 1.5], PD = 83.4\%, BF = 0.64$. There was moderate evidence of an interaction such that older children were more likely to stop early in the Dense condition $OR = 3.62, 95\%CI = [1.07, 12.21], PD = 98.1\%, BF = 5.3$. For comparison, our random intervention baseline simulations produced test sequences

For Review Only

Table S2

Counts and Percentage of Children Stopping Early and Number of Unnecessary Tests Performed, and Average Number of Total Tests Performed (SD).

Age group	Condition	N Participants	Stopped Early	Tested Unnecessarily	N tests
Younger	Sparse	21	10 (47%)	1 (5%)	3.00 ± 1.94
	Dense	25	12 (48%)	1 (4%)	3.52 ± 1.33
Older	Sparse	26	3 (12%)	1 (4%)	2.81 ± 1.30
	Dense	20	13 (59%)	0 (0%)	3.00 ± 1.52

that rarely resolved all uncertainty by the time participants made their judgment, effectively being classified as stopping early 60% of the time in the Sparse condition, and 89% of the time in the Dense condition.

For Review Only
